# État des lieux de la vaccination contre la Covid-19 du personnel du Centre hospitalier universitaire de Tengandogo, Ouagadougou-Burkina Faso, juin à décembre 2021

**DOI:** 10.48327/mtsi.v5i2.2025.625

**Published:** 2025-06-03

**Authors:** Wedminère Noélie ZOUNGRANA-YAMEOGO, David KANGOYE, Issa OUEDRAOGO, Yassia BAMOGO, Abdoulaye SO, Arielle Rita BELEM, Alban Michel BASSOLE, Idrissa SANOU

**Affiliations:** 1 Département de santé publique, CHU de Tengandogo, Ouagadougou, Burkina Faso; 2 Institut national de santé publique, Ouagadougou, Burkina Faso; 3 Direction de la prévention par la vaccination, Ministère de la santé, Ouagadougou, Burkina Faso; 4 Service des urgences polyvalentes, CHUde Tengandogo, Ouagadougou, Burkina Faso; 5 Direction de la qualité, CHU de Tengandogo, Ouagadougou, Burkina Faso; 6 Services de médecine et de spécialités médicales, CHU de Tengandogo, Ouagadougou, Burkina Faso; 7 Département du laboratoire et de la pharmacie hospitalière, CHU de Tengandogo, Ouagadougou, Burkina Faso

**Keywords:** Covid-19, Vaccination, Observance, Personnel, Hôpital, Burkina Faso, Afrique subsaharienne, COVID-19, Vaccination, Compliance, Staff, Hospital, Burkina Faso, Sub-Saharan AfricaIntroduction

## Abstract

**Introduction:**

Selon l'OMS, fin 2021, 278 millions de cas confirmés de Covid-19 ont été enregistrés dans le monde avec environ 5,4 millions de décès. Au Burkina Faso, ce sont 17 632 cas avec 318 décès qui ont été enregistrés. La vaccination est une des stratégies mise en place pour lutter contre cette pandémie. L'objectif de l'étude était de faire un état des lieux de la vaccination contre la Covid-19 chez le personnel d'un hôpital de référence du Burkina Faso.

**Méthodes:**

Nous avons conduit une étude descriptive allant du 2 juin 2021 au 31 décembre 2021 au CHU de Tengandogo (CHU-T). Elle a concerné l'ensemble du personnel. Les informations ont été obtenues par entretien face à face, téléphonique et par questionnaires autoadministrés. Les variables quantitatives ont été décrites en utilisant la moyenne et l'écart type et les variables qualitatives en utilisant la proportion. Toutes les personnes vaccinées ont répondu au questionnaire. Les personnes non vaccinées étaient des volontaires.

**Résultats:**

Au total 31 % (174/559) des agents de santé ont été vaccinés; leur âge moyen était de 41 ± 8 ans. Les hommes représentaient 55 % (94/174) des agents vaccinés. Parmi les agents, les principaux profils étaient représentés par 62 (35,6 %) médecins, 62 (35,6 %) infirmiers, 9 (5,2 %) garçons et filles de salle. La principale raison d'acceptation de la vaccination était la protection contre la maladie enregistrée chez 132 agents soit 76 %. Les sujets non vaccinés ayant accepté de participer à l'étude étaient au nombre de 134. L'âge moyen était 32,8 ±7,3 ans. La proportion de femmes était de 60 %. L'incertitude concernant l'efficacité des vaccins a été la principale raison de la non-vaccination notée chez 106 agents 79 % (106/134). Des évènements indésirables mineurs ont été signalés chez 136 agents vaccinés soit 78 %. Aucun évènement indésirable grave n'a été signalé. Quatre agents vaccinés ont développé la Covid-19 au cours de la période de l'étude.

**Conclusion:**

La proportion des sujets vaccinés était faible. Des interventions visant à améliorer l'adhésion des agents de santé à la vaccination devraient être développées.

## Abstract (en)

**Introduction:**

According to the WHO, 278 million confirmed cases of COVID-19 had been recorded worldwide by the end of 2021, resulting in approximately 5.4 million deaths. In Burkina Faso, 17,632 cases and 318 deaths were recorded. Vaccination is one of the strategies implemented to control the pandemic. This study aimed to evaluate the status of the COVID-19 vaccination program among staff at a referral hospital in Burkina Faso.

**Methods:**

From June 2 to December 31, 2021, we conducted a descriptive study at Tengandogo University Hospital (CHU-T). The study involved all staff members. Information was obtained through face-to-face and telephone interviews and self-administered questionnaires. Quantitative variables were described using the mean and standard deviation, while qualitative variables were described using proportions. All vaccinated individuals responded to the questionnaire. Unvaccinated individuals were volunteers.

**Results:**

A total of 31% (174 out of 559) of healthcare workers were vaccinated, with a mean age of 41 ± 8 years. Men accounted for 55% (94/174) of the vaccinated workers. The main worker profiles were 62 doctors (35.6%), 62 nurses (35.6%), and nine ward boys and girls (5.2%). The main reason for accepting the vaccine was protection against the disease, reported by 132 workers (76%). There were 134 unvaccinated subjects who agreed to participate in the study. Their average age was 32.8 ± 7.3 years. The proportion of women was 60%. Uncertainty about vaccine effectiveness was the main reason for not getting vaccinated, cited by 106 workers (79%). Minor adverse events were reported by 136 of the vaccinated workers (78%). No serious adverse events were reported. Four vaccinated workers developed symptomatic SARS-CoV-2 infection during the study period.

**Conclusion:**

The proportion of vaccinated subjects was low. Interventions to improve healthcare workers’ adherence to vaccination should be developed.

## Introduction

Selon l'OMS, le 26 décembre 2021, 278 millions de cas confirmés de Covid-19 ont été enregistrés dans le monde avec environ 5,4 millions de décès [6]. Le Burkina Faso a notifié ses premiers cas de Covid-19 le 9 mars 2020 [10]. Á la date du 2 janvier 2022, le Burkina Faso avait enregistré 18 637 cas dont 7 662 femmes et 13 063 hommes avec un cumul de 375 décès soit une létalité de 1,8 % [10]. Dans le but de lutter contre cette pandémie, diverses mesures de prévention ont été utilisées. Les plus importantes étaient les mesures de distanciation physique, l'utilisation des masques et la fermeture de certains lieux publics. La vaccination faisant partie des moyens de prévention a été une stratégie qui a été développée après la mise au point de vaccins homologués. Les premiers objectifs de la campagne de vaccination contre le SARS-CoV-2 étaient de réduire la mortalité, les formes graves de la Covid-19 et la tension sur le système de santé. La vaccination permet en outre de contrôler la circulation du virus et de contenir l'épidémie à partir du taux de couverture requis et limite l'apparition de nouveaux variants plus transmissibles. Á la date du 18 février 2021, au moins 7 vaccins différents avaient été mis à disposition des pays par l'intermédiaire de trois plateformes pendant que d'autres vaccins étaient en phases d'essais cliniques [15]. L'efficacité de la vaccination contre la Covid-19 varie d'un vaccin à l'autre et, pour certains d'entre eux, elle varie selon le schéma vaccinal, en fonction du nombre de doses. Pour quelques vaccins, l'efficacité est meilleure chez le sujet jeune, mais elle peut être améliorée par plusieurs doses lorsque le sujet est âgé [13].

De juin à décembre 2021, les autorités du Burkina Faso ont entrepris des campagnes de vaccination pour faire face à la Covid-19. Quatre types de vaccins ont été introduits. Le CHU-T a initié la vaccination de son personnel le 8 juin 2021 sous forme de campagnes de vaccinations gratuites. Elles ont commencé avec le vaccin Astra Zeneca puis le Johnson & Johnson. Les vaccins Pfizer et Sinopharm ont été utilisés plus tard.

Fin octobre 2021, 325 119 (2 % de la population) personnes avaient été vaccinées sur le plan national [2, 11]. La présente étude a pour objectif de faire un état des lieux de la vaccination du personnel du CHU-T.

## Méthodes

Il s'agit d'une étude descriptive transversale conduite du 2 juin au 31 décembre 2021 au Burkina Faso, pays situé en Afrique de l'Ouest. Le dernier recensement général de la population et de l'habitat a dénombré 20 487 979 habitants [7]. L'étude s'est déroulée au sein du CHU-T, un hôpital de troisième niveau selon la pyramide sanitaire du Burkina Faso. L'hôpital est situé dans la commune de Komsilga, banlieue de Ouagadougou, province du Kadiogo, région du centre du Burkina Faso. La majorité de ses patients proviennent de Ouagadougou, capitale politique et administrative du Burkina Faso. Elle compte 4 centres hospitalo-universitaires dont le CHU-T. Sa population était estimée en 2021 à environ 2,5 millions d'habitants, soit 45 % de la population urbaine. Son climat est de type sahélien avec une saison pluvieuse par an. Sa population est en majorité musulmane. Le commerce de détail constitue sa principale activité.

Le CHU-T a officiellement ouvert ses portes au public le 1^er^ septembre 2011. La prise en charge des urgences ainsi que les hospitalisations ont commencé le 8 octobre 2012. Sa vocation est d’être un hôpital de référence du Burkina Faso, voire de la sous-région Ouest africaine et au-delà. L'hôpital a une capacité de 600 lits, mais 325 étaient en service en 2021. Le nombre de consultations s’élevait à 32 555 en 2021 et le nombre d'admissions à 10 477. La vaccination contre la Covid-19 a commencé le 8 juin 2021 au CHU-T sous la forme de campagnes de vaccination et de juin à décembre 2021, 6 campagnes y ont été réalisées.

La population d’étude était constituée des agents de l'hôpital, tous profils confondus.

Les variables de l'étude étaient regroupées dans les rubriques suivantes : les raisons de la vaccination ou de la non-vaccination, le type de vaccin utilisé, les évènements indésirables chez les sujets vaccinés, leur délai d'apparition et le comportement de l'entourage suite aux évènements indésirables ressentis chez les personnes vaccinées.

Les informations sur les raisons des vaccinations ont été recueillies auprès des agents par entretien en face à face ou téléphonique à l'aide d'un questionnaire. Les entretiens concernaient ceux qui avaient accepté de se faire vacciner (Annexe 1). L'enquête a été menée par deux médecins, un généraliste et un épidémiologiste. Pour ceux qui n'avaient pas accepté la vaccination, les questionnaires leur ont été remis pour une auto-administration (Annexe 2). Pour le schéma vaccinal à deux doses, l'étude s'est intéressée à la première dose. Pour éviter les biais de mémoire, les entretiens ont été effectués dans les deux semaines qui ont suivi la vaccination. En outre, pendant la durée de l'étude (juin à décembre 2021), nous avons intensifié le dépistage de la Covid-19 par PCR auprès du personnel vacciné, en envoyant régulièrement des messages dans le groupe WhatsApp où se trouvent les contacts téléphoniques de tous les agents de l'hôpital. Les résultats des tests PCR du personnel ont été examinés. Nous avons pu obtenir l'information en lien avec l'apparition de la maladie entre juin et décembre 2021.

Nous avons réalisé un échantillonnage exhaustif de tous les agents qui avaient accepté la vaccination. Pour ceux qui n’étaient pas vaccinés, l'échantillonnage était volontaire avec auto-administration du questionnaire.

La principale variable était le nombre d'agents vaccinés, ce qui nous a permis de calculer la couverture vaccinale. Les données ont été traitées et analysées avec le logiciel STATA version 15. La moyenne et l'écart type ont été utilisés pour décrire les variables quantitatives. La proportion a été utilisée pour décrire les variables qualitatives. Nous avons obtenu l'autorisation de la direction générale du CHU-T pour mener l'enquête auprès des agents de l'hôpital. Le consentement oral des agents de l'hôpital a été obtenu avant de les inclure dans l'étude. Toutes les dispositions ont été prises en compte pour préserver la confidentialité des données des participants. La fiche de collecte était anonyme.

## Résultats

À la date du 31 décembre 2021, 559 agents tous profils confondus travaillaient au CHU-T dont 61 % étaient de sexe féminin. Parmi eux, on comptait 123 médecins (22 %), 251 infirmiers (45%), 36 sages femmes (6 %), 91 filles et garçons de salle (16 %) et 10 pharmaciens (2 %). Le reste du personnel au nombre de 48 soit 9 % était constitué du personnel administratif et du personnel d'appui technique.

Au total, 174 agents (27,4 %) ont été vaccinés entre le 2 juin et le 31 décembre (Tableau I). L'âge moyen des agents vaccinés était 41,3 ans ± 7,9. Les hommes étaient au nombre de 94 (54 %) et les femmes 80 (46 %). Les vaccins administrés étaient Astra Zeneca chez 110 agents (63 %), Johnson & Johnson chez 63 agents (36 %), et Pfizer (pour 1 personne). Au total, 47 agents vaccinés ont signalé des comportements de refus de se faire vacciner de la part de leur entourage suite aux évènements indésirables ressentis, et 89 agents (22 %) aucun comportement. L'information était non applicable chez 38 agents (27 %).

Les principales raisons d'acceptation de la vaccination étaient la protection contre la maladie (n = 132; 76 %), un voyage à l'étranger (n = 23; 33 %) et le fait de s'y sentir obligé (n = 10; 6 %). Les médecins vaccinés étaient 62 sur 123 médecins (50 %) et les infirmiers 62 sur 251 infirmiers (25 %). L'effectif du personnel vacciné ou non interrogé est précisé dans le Tableau I.

**Tableau I:** Effectifs des agents vaccinés ou non interrogés selon le profil de juin à décembre 2021 au CHU-T

Fonction	Effectif total (N)	Vaccinés, n (%)	Vaccinés interrogés	Non vaccinés n (%)	Non vaccinés interrogés n (% non vaccinés)
Médecins	123	62 (50)	62	61 (50)	55 (90)
Infirmiers	251	62 (25)	62	189 (75)	31 (16)
Pharmaciens	10	5 (50)	5	5 (50)	2 (40)
Sages-femmes	36	7 (19)	7	29 (80)	10 (34)
Filles et garçons de salle	91	9 (9)	9	82 (90)	5 (6)
Agents des ressources humaines (DRH)	8	8 (100)	8	0 (0)	0 (0)
Manipulateurs en radiologie	4	4 (100)	4	0 (0)	0 (0)
Agents de kinésithérapie	4	2 (50)	2	2 (50)	0 (0)
Techniciens de laboratoire	17	3 (18)	3	14 (82)	3 (21)
Informaticiens	2	2 (100)	2	0 (0)	0 (0)
Secrétaires	14	3 (21)	3	11 (79)	7
Standardistes	2	1 (50)	1	0 (0)	0 (0)
Administrateurs affaires sociales	2	1 (50)	1	1 (50)	0 (0)
Agents de la buanderie	6	1 (17)	1	5 (83)	0 (0)
Comptables	8	2 (25)	2	6 (75)	1 (13)
Caissiers	13	1 (8)	1	12 (92)	0 (0)
Chauffeurs	6	1 (1)	1	5 (83)	0 (0)

Au total, 78 % (136/174) des agents vaccinés ont signalé des évènements indésirables après avoir été vaccinés dont 89 % (121/136) dans les 24 heures qui ont suivi la vaccination, 10 % (14/136) dans la semaine suivante et une personne dans les deux semaines. Parmi les personnes qui ont signalé des évènements indésirables, 88 (soit 75 %) avaient été vaccinées avec Astra Zeneca et 47 (soit 35 %) avec le Johnson & Johnson. Aucun évènement indésirable n'a été enregistré chez l'agent qui a reçu le vaccin Pfizer.

Le Tableau II présente les principaux évènements indésirables selon le type de vaccin reçu. En tout 2,3 % (4/174) des agents vaccinés ont été testés positifs à la Covid-19 par la PCR réalisée au laboratoire du CHU-T. Trois d'entre eux, vaccinés avec le vaccin Johnson & Johnson, ont développé la Covid-19 respectivement à 30, 66, et 74 jours après la vaccination. L'agent vacciné avec le vaccin Astra Zeneca a été testé positif 174 jours après avoir reçu le vaccin. L'incidence de la Covid-19 chez les agents non vaccinés n'est pas connue. Nous avons pu avoir les informations sur les raisons de non-acceptation de la vaccination chez 134 personnes soit 29 % (134/462) du personnel non vacciné, 60 % (80/134) étaient de sexe féminin. L'âge moyen des agents non vaccinés interrogés était de 33 ans ±8. Pour ce qui concerne les profils des enquêtés non vaccinés, l'information était disponible pour 126 personnes sur les 134 (8 informations manquantes). Les médecins étaient au nombre de 55 (44 %), les infirmiers 31 (28 %), les filles/garçons de salle 10 (8 %), les secrétaires 7 (8 %), les sages-femmes 5 (4 %), les aides-soignants 4 (3 %), infirmiers attachés de recherche 4 (3%), les techniciens de laboratoire 3 (2 %), les pharmaciens 2 (2 %), les techniciens d’état en génie sanitaire 2 (2%), les agents des ressources humaines 1 (2%), les agents comptables 1 (1%), autre non précisé 1(1%).

**Tableau II:** Principaux évènements indésirables enregistrés selon Johnson and Johnson et AstraZeneca reçu chez les agents vaccinés contre la Covid-19 de juin à décembre 2021 au CHU-T

Évènements indésirables	Modalités	J&J		Astra Zeneca	
n	%	n	%
Courbatures	Oui	22	35	66	60
	Non	41	65	44	40
Céphalées	Oui	15	24	54	51
	Non	48	76	56	49
Fièvre	Oui	9	14	59	54
	Non	54	86	51	46
Douleur au point d'injection	Oui	23	36	46	26
	Non	40	63	128	74
Sensation de mal être	Oui	22	35	26	24
	Non	41	65	84	76
Vertiges	Oui	5	8	9	8
	Non	58	92	101	92
Vomissements	Oui	0	0	4	4
	Non	0	0	106	96
Nausées	Oui	1	2	8	7
	Non	62	98	102	93
Épistaxis	Oui / Yes	0	0	1	1
	Non	0	0	173	99

Les principales raisons de la non-acceptation de la vaccination selon les profils sont présentées dans le Tableau III. D'autres raisons ont été également recueillies mais ces raisons n’étaient pas associées à des profils particuliers car l'information manquait au niveau de ce champ. Ces raisons étaient les suivantes : décès post vaccination (n = 1), manque d'expérience pour la fabrication des vaccins (n = 1), vaccin non disponible au moment du pic (n = 1), période de temps d'immunité réduit (n = 1), pas d'information sur le lieu de fabrication de la vaccination (n = 1), manque de confiance aux pays producteurs (= 1), temps de pharmacovigilance court (n = 1), pas d'information sur la fertilité chez les femmes (n = 1).

**Tableau III:** Raisons de la non-acceptation de la vaccination contre la Covid-19 chez 126 membres du personnel (tous profils confondus) du CHU-T de juin à décembre 2021

Raison de la non-vaccination	Tous profils (N = 126)	%	Médecins+ Pharmaciens (N = 57)	% (n /N)	Infirmiers + Sages-femmes + Aides-soignants + Techniciens de laboratoire (N = 43)	% (n /N)	*Autres agents (N = 26)	% (n /N)
Incertitudes concernant l'efficacité	106	84	46	81	35	81	20	77
Incertitudes concernant la sécurité	99	79	48	84	28	65	18	69
Débats contradictoires entre chercheurs sur les vaccins	92	73	41	72	31	72	12	46
Convenance personnelle	89	71	40	70	24	56	21	81
Débats contradictoires entre chercheurs sur la maladie	79	63	34	60	28	65	15	58
Évènements indésirables ressentis par les proches	69	55	26	46	26	61	13	50
Cas survenus chez des sujets vaccinés	54	41	11	19	28	65	13	50
Multiplicité vaccins	54	43	27	47	18	42	6	23
Pas confiance au gouvernement	41	33	20	35	11	26	7	27
Faible incidence au Burkina Faso	40	32	19	33	10	23	6	23
Conservation douteuse des vaccins	38	30	17	30	11	26	7	27
Informations contradictoires sur les réseaux sociaux	28	22	9	16	11	26	15	58
Faible létalité au Burkina	34	27	19	33	5	12	5	19
Allergies aux vaccins	23	18	6	11	13	30	4	15
Déjà vacciné ou malade	18	14	5	9	6	14	6	23
Non croyance à la maladie	14	11	5	9	5	12	3	12
Présence de grossesse	9	7	1	2	3	7	4	15
Vaccins provenant des pays étrangers	37	29	15	26	11	26	9	35

*Autres agents : (filles et garçons de salle (n = 10), agents des ressources humaines (n = 1), agent de la comptabilité (n = 1), secrétaires (n = 7), techniciens d’état en génie sanitaire (n = 2), attachés de santé en recherche (n = 4), autre profil non précisé (n = 1)

## Discussion

Cette étude nous a permis de dresser un état des lieux de la vaccination contre la Covid-19 chez les agents du CHU-T et de déterminer les raisons de l'acceptation ou de la non-acceptation de la vaccination.

Le fait que l'étude ait été réalisée dans un seul hôpital, même si celui-ci était l'hôpital de référence pour la prise en charge des patients atteints de la Covid-19 au début de la pandémie en 2020, ne permet pas de généraliser les résultats à l'ensemble des établissements sanitaires du Burkina Faso. Une autre limite de l'étude est que nous n'avons pas enregistré les cas de Covid-19 chez les personnes non vaccinées durant la période de l'étude, ce qui aurait pu servir d'argument pour motiver le personnel à se faire vacciner. Par ailleurs, le faible nombre de sujets interrogés sur les raisons de la non-vaccination constitue une limite non seulement sur la représentativité de l'échantillon mais aussi sur l'extrapolation des résultats de l'étude. L'auto-administration du questionnaire par les personnes non vaccinées pourrait également biaiser les résultats de l'étude car ceux-ci pourraient fournir des réponses inexactes suite à une mauvaise compréhension des questions surtout que ces questions étaient fermées. Néanmoins, les résultats de cette étude donnent des indications précieuses sur les motivations de vaccination ou non vaccination des personnels hospitaliers.

La faible proportion du personnel vacciné au sein du CHU-T, témoigne d'une réticence chez près de 70 % des agents de santé. Cette proportion de réticence est au-dessus de celle trouvée par Khamis *et al.* dans une étude analytique sur l'hésitation vaccinale réalisée chez 433 travailleurs de la santé à Oman au Moyen Orient en 2021 [9]. En effet, dans le contexte du Burkina Faso, beaucoup de préjugés ont accompagné l'épidémie de Covid-19. Des citoyens ont pensé que la Covid-19 était une maladie des personnes riches du fait que les premières personnes chez qui la maladie avait été diagnostiquée revenaient de voyage après un séjour à l'étranger notamment en Europe. Pour d'autres, le sujet africain de peau noire était comme immunisé contre la maladie [5, 16]. Certains considéraient qu'il s'agissait d'une maladie banale comme toutes autres pneumopathies virales (comme la grippe saisonnière), au vu de la très faible incidence et létalité, comparé à ce qui était décrit dans les autres continents [4, 12]. Les capacités de prise en charge des malades étaient bien souvent saturées [3, 5], ce qui a certainement contribué à inciter le personnel soignant à se faire vacciner pour ne pas être contaminé.

La proportion des agents de santé vaccinés était faible, davantage chez les infirmiers que chez les médecins alors que ces agents sont en première ligne de la riposte contre la Covid-19 dans les hôpitaux. Leur risque de contamination est beaucoup plus élevé en raison du contact permanent et parfois prolongé avec les patients [8].

La vaccination contre la Covid-19 a été proposée comme stratégie de lutte, mais cette stratégie a été diversement acceptée au sein de la population, des professionnels de santé, des chercheurs (santé publique, immunologistes, biologistes, microbiologistes, etc.) partout dans le monde. Dans notre étude, la principale raison d'acceptation de la vaccination était la crainte d’être contaminé, raison également retrouvée par Paquin *et al.* dans une étude analytique réalisée chez 342 agents de santé en Côte d'Ivoire en 2021 [16]. L'obligation de la vaccination pour raison de voyage a occupé une place importante parmi les motivations à la vaccination. Pendant la pandémie, la vaccination était une des conditions indispensables d'entrée dans plusieurs pays.

De façon générale, les raisons communes de non-acceptation à la vaccination chez les médecins, les infirmiers et les autres agents portaient sur l'incertitude concernant l'efficacité, la sécurité et les débats contradictoires des chercheurs sur les vaccins et sur la maladie en elle-même. Cependant, les évènements indésirables ressentis par un proche vacciné, la survenue de Covid-19 chez des sujets vaccinés ont été majoritairement évoqués dans le groupe des infirmiers et autres agents, ce qui n'est pas le cas chez les médecins. Ce comportement de refus suite à ces informations est probablement dû au fait que les sources d'informations n’étaient pas fiables, beaucoup d'informations provenant des réseaux sociaux ou des sites Internet non certifiés. Les autorités politiques à travers le ministère de la Santé et le ministère en charge de la Recherche devraient dès les premiers moments de l'épidémie sensibiliser les acteurs de la santé sur les sources d'informations scientifiques fiables et veiller également à ce que les sources d'informations nationales soient d'origine institutionnelle. Des sondages rapides pro-actifs des populations cibles pour mieux cerner leurs préoccupations et leurs besoins en information fourniraient des données utiles pour calibrer la communication officielle à l'adresse des vaccinosceptiques et antivaccins. Par ailleurs les sensibilisations, les formations et les stratégies de prévention devraient être spécifiques aux différents profils.

Les raisons de la non-vaccination telles que l'inquiétude concernant l'efficacité et la sécurité ont été décrites par d'autres auteurs [2, 16]. La crainte des évènements indésirables, l'incertitude ressentie par les professionnels de santé sur le manque de transparence sur les essais cliniques et les protocoles de recherche et sur l'efficacité des vaccins ont été décrites par Touré *et al.* dans une étude descriptive réalisée chez 303 usagers de deux structures de santé de la commune de Youpougon en Côte d'Ivoire [18]. La peur d'un effet indésirable des vaccins sur une grossesse ou sur la survenue de grossesse est également décrite par Touré *et al.* [18].

Les évènements indésirables mineurs tels que les céphalées, les douleurs au point d'injection et la fièvre couramment ressentis et décrits dans plusieurs études sont fréquents et témoignent d'une réaction immunitaire normale [14]. Nous n'avons noté aucun évènement indésirable grave chez les agents vaccinés. Ceci pourrait s'expliquer par le fait que notre enquête était transversale et que l'effectif de la population d’étude était faible. Trois personnes sur 110 (2,7 %) vaccinées avec le vaccin Johnson & Johnson et une personne sur 63 (1,6 %) vaccinées avec le vaccin AstraZeneca ont été testées positives après avoir présenté des signes mineurs de l'infection par la Covid-19. Des cas de Covid-19 post vaccinations ont été décrits par d'autres auteurs [1, 17].

## Conclusion

Cette étude nous a permis de faire un bilan sur l'acceptation de la vaccination contre la Covid-19 dans notre hôpital, de recenser les raisons de l'acceptation et de la non-acceptation de la vaccination et de faire le point sur les évènements indésirables rapportés après la vaccination par les vaccins Astra Zeneca et Johnson & Johnson. La proportion du personnel de santé ayant accepté de se faire vacciner est faible pour les agents de première ligne qui sont impliqués dans la lutte contre la Covid-19 et contre les autres maladies infectieuses. Des interventions visant à améliorer l'adhésion chez ce personnel de première ligne doivent être développées.

## Financement de l'étude

Cette étude n'a reçu aucun financement.

## Remerciements

Nous remercions le Dr Souleymane Kaboré qui a significativement contribué à la réalisation de cette étude, mais qui est décédé malheureusement après l'élaboration de la version finale du manuscrit. Nous lui rendons un grand hommage.

## Contributions des auteurs et autrices

Noélie Zoungrana-Yameogo est le concepteur de l'étude. Elle a participé à la collecte des données et a réalisé l'analyse et l'interprétation des données. Souleymane Kabore a participé à la rédaction du manuscrit final. Les docteurs David Kangoye, Issa Ouedraogo, Yassia Bamogo, Abdoulaye So, Arielle Rita Belem et Alban Michel Bassolé, ont lu et apporté des observations à la version définitive du manuscrit. Le Professeur Idrissa Sanou a approuvé la publication du manuscrit.

## Conflits d'intérêts

Les auteurs ne déclarent aucun conflit d'intérêts.

## Annexe 1 : Questionnaire adressé aux agents vaccinés

Bonjour,

Je suis docteur …, nous souhaiterons vous interroger sur la vaccination contre la Covid-19 dans le cadre d'une étude dont l'objectif est de faire un état des lieux de la vaccination contre la Covid-19 des agents du CHU-T. Nous avons obtenu votre contact à partir du registre des agents vaccinés. La publication des résultats se fera de façon anonyme, vous êtes libre de participer ou de ne pas participer à l'étude.

**1. Données générales**

Âge : /__/ ans Sexe : M /__/ F /_ /

Qualification : Médecin /__/ Pharmacien /__/ Infirmier(e) /__/ Technicien de laboratoire /__/

Manipulateur d’état en radiologie /__/ Aide-soignant(e) /__/ Fille/Garçon de salle /__/ Sage-femme /__/

Agent ressource humaine /__/ Communicateur /__/ Chauffeur /__/ Buandier /__/ Vigile /__/

Informaticien /__/ Kinésithérapeute /__/ Secrétaire /__/ Autres /__/ Si autre à préciser__

**2. Qu'est-ce qui vous a motivé pour la vaccination ?**

Peur de faire la maladie /__/ se sentir obligé /__/ l'exemple d'un collègue /__/

Obligation du supérieur hiérarchique /__/ L'exemple du supérieur hiérarchique /__/

Contribution à la science /___ / L'exemple d'un membre de la famille /___ / Voyage /___ /

Autres /___ / Si autre à préciser __

**3. Type de vaccination de vaccin**

Type de vaccin reçu : Astra Zenaca /__/ Johnson and Johnson /__/ Autre /__/

**4. Avez-vous eu des évènements indésirables après la vaccination ?**

Oui /__ / Non /__/

Si oui

1) Céphalées Oui /__/ Non /__/

2) Fièvre Oui /__/ Non /__/

3) Courbatures Oui /__/ Non /__/

4) Douleurs au point d'injection Oui /__/ Non /__/

5) Rougeurs Oui /__/ Non /__/

6) Vertiges Oui /__/ Non /__/

7) Sensation de mal être Oui /__/ Non /__/

8) Nausées Oui /__/ Non /__/

9) Vomissements Oui /___ / Non /_ /

10) Thromboses Oui /__/ Non / /

11) Myocardites Oui /__/ Non / / Autres /__/

**5. Quelle était le délai d'apparition des évènements indésirables ?**

Immédiatement /__ / Jours /__ / mois /__ /

**6. Réaction de votre entourage par rapport aux évènements indésirables**

Refus de se faire vacciner /_ / Aucune réaction /___ / NA si pas d'effets secondaires /___ /

## Annexe 2 : Questionnaire adressé aux agents non vaccinés (volontaires)

Bonjour, je suis docteur …, nous souhaiterons recueillir des informations sur les raisons de la non-vaccination contre la Covid-19 auprès des agents non vaccinés du CHU-T. Votre participation à cette enquête à travers le remplissage de cette fiche est totalement volontaire, vos données anonymes et les résultats qui seront présentés seront anonymes.

Merci pour votre participation.

**1. Données générales**

Âge : /__/ ans Sexe : M /__/ F /__/

Qualification : Médecin /__/ Pharmacien /__/ Infirmier(e) /__/ Technicien de laboratoire /__/

Manipulateur d’État en radiologie /__/ Aide-soignant(e) /__/ Fille/Garçon de salle /__/ Sage-femme /__/

Communicateur /__/ Chauffeur /__/ Buandier /__/ Vigile /__/ Informaticien /__/ Électricien /__/

Agent DRH /__/ Kinésithérapeute /__/ Secrétaire /__/ Agent DFC /__/

Autres /__/ Si autre à préciser__

**2. Les raisons de non-vaccination, plusieurs choix sont possibles**

1) Incertitude concernant l'efficacité Oui /__/ Non /__/

2) Incertitude concernant la sécurité Oui /__/ Non /__/

3) Multiplicité des vaccins Oui /__/ Non /__/

4) Non croyance à la maladie Oui /__/ Non /__/

5) Faible incidence (pas beaucoup de cas au Burkina Faso) Oui /__/ Non /__/

6) Faible létalité (pas beaucoup de morts au Burkina Faso) Oui /__/ Non /__/

7) Évènements indésirables ressentis par un proche Oui /__/ Non /__/

8) Vaccins provenant d'autres horizons Oui /__/ Non /__/

9) Débats contradictoires entre chercheurs sur la maladie Oui /__/ Non /__/

10) Débats contradictoires entre chercheurs sur les vaccins Oui /__/ Non /__/

11) Doutes sur la conservation des vaccins (température de conservation) Oui /__/ Non /__/

12) Allergies aux vaccins Oui /__/ Non /__/

13) Grossesse Oui /__/ Non /__/

14) Déjà fait la Covid-19 Oui /__/ Non /__/

15) Informations contradictoires sur les réseaux sociaux Oui /__/ Non /__/

16) Non confiance au gouvernement Oui /__/ Non /__/

17) Convenance personnelle Oui /__/ Non /__/

18) Des cas de Covid-19 signalés chez des sujets déjà vaccinés Oui /__/ Non /__/

19) Autres à préciser…….
