# Supplementary material for: Status of COVID-19 vaccination among staff at the Tengandogo University Hospital Center, Ouagadougou, Burkina Faso, June to December 2021
Source: Med Trop Sante Int. 2025 Jun 3;5(2):mtsi.v5i2.2025.625. [Article in French] doi: 10.48327/mtsi.v5i2.2025.625 (PMC12308574; doi:10.48327/mtsi.v5i2.2025.625)
Supplement: Related texts

# Research article: Status of COVID-19 vaccination among staff at the Tengandogo University Hospital Center, Ouagadougou, Burkina Faso, June to December 2021

## Introduction

As of December 26, 2021, the WHO reported 278 million confirmed cases of and approximately 5.4 million deaths from COVID-19 worldwide [6]. Burkina Faso reported its first cases on March 9, 2020. As of January 2, 2022, the country had recorded 18,637 cases, including 7,662 women and 13,063 men. There were a total of 375 deaths, representing a case fatality rate of 1.8%. Various preventive measures have been implemented to combat the pandemic. The most important measures were physical distancing, mask-wearing, and closing certain public places. Vaccination as a means of prevention was a strategy developed after approved vaccines were available. The initial objectives of the SARS-CoV-2 vaccination campaign were to reduce mortality and severe cases of the disease, as well as pressure on the healthcare system. Vaccination also helps control the spread of the virus, contain the epidemic once the required coverage rate is reached, and limit the emergence of new, more transmissible variants. As of February 18, 2021, at least seven different vaccines had been made available to countries through three platforms, while other vaccines were in clinical trials. The effectiveness of vaccines against COVID-19 varies depending on the vaccine and, for some, the vaccination schedule and number of doses. Some vaccines are more effective in young people, but multiple doses can improve effectiveness in older people [13]. From June to December 2021, the authorities in Burkina Faso launched vaccination campaigns to contain the virus. Four types of vaccines were introduced. On June 8, 2021, the CHU-T began vaccinating its staff through free vaccination campaigns. They began with the AstraZeneca vaccine, followed by the Johnson & Johnson vaccine. The Pfizer and Sinopharm vaccines were used later. As of the end of October 2021, 325,119 people (2% of the population) had received the vaccine nationwide [2, 11]. This study aims to provide an overview of CHU-T staff vaccination.

## Methods

This descriptive, cross-sectional study was conducted from June 2 to December 31, 2021, in Burkina Faso, a country in West Africa. The most recent general population and housing census recorded 20,487,979 inhabitants [7].

The study took place at the CHU-T, which is a tertiary care hospital according to Burkina Faso's healthcare pyramid. It is located in the Komsilga municipality, a suburb of Ouagadougou in the Kadiogo province, which is in central Burkina Faso. Most of its patients come from Ouagadougou, the political and administrative capital of Burkina Faso. Ouagadougou has four university hospitals, including the CHU-T. In 2021, its population was estimated at around 2.5 million, representing 45% of the urban population. The city has a Sahelian climate with one rainy season per year. The majority of the population is Muslim. Retail trade is the main activity.

The CHU-T officially opened to the public on September 1, 2011. Emergency care and hospitalizations began on October 8, 2012. The hospital's mission is to serve as a referral hospital for Burkina Faso, the West African subregion, and beyond. The hospital has 600 beds, but only 325 were in use in 2021. The number of consultations stood at 32,555 in 2021, and the number of admissions at 10,477.

The CHU-T began administering the first of six vaccination campaigns on June 8, 2021, to restrain the spread of the SARS-CoV-2 virus.

The study population consisted of hospital staff from all departments.

The study variables were grouped into the following categories: reasons for or against vaccination, type of vaccine used, adverse events experienced by vaccinated individuals, time of onset of these events, and the behavior of those around them following these events.

Information on reasons for vaccination was collected from staff members via face-to-face or telephone interviews using a questionnaire. Interviews were conducted with individuals who had agreed to be vaccinated (see Appendix 1). Two physicians, a general practitioner and an epidemiologist, conducted the survey. For those who did not agree to be vaccinated, the physicians provided them with a questionnaire to fill out themselves (Appendix 2). The study focused on the first dose for the two-dose vaccination schedule. To avoid memory bias, the interviews were conducted within two weeks of vaccination. During the study period (June to December 2021), we also intensified PCR screening for SARS-CoV-2 among vaccinated staff by regularly sending messages to a WhatsApp group containing the phone numbers of all hospital staff. We reviewed the results of the staff PCR tests. We obtained information related to the onset of the disease between June and December 2021.

We conducted comprehensive sampling of all staff who agreed to be vaccinated. For those who were not vaccinated, sampling was voluntary and involved self-administered questionnaires.

The main variable was the number of vaccinated staff, which enabled us to calculate vaccination coverage. We processed and analyzed the data using STATA version 15 software. We used the mean and standard deviation to describe quantitative variables. Proportions were used to describe qualitative variables.

We obtained authorization from CHU-T's senior management to conduct the survey with hospital staff. We obtained the verbal consent of the staff before including them in the study. All measures were taken to preserve the confidentiality of the participants’ data. The data collection form was anonymous.

## Results

As of December 31, 2021, 559 staff members of various profiles were employed at the CHU-T, 61% of whom were female. Of those, 123 were doctors (22%), 251 were nurses (45%), 36 were midwives (6%), 91 were ward assistants (16%), and 10 were pharmacists (2%). The remaining 48 staff members (9%) were administrative and technical support staff.

A total of 174 staff members (27.4%) received the vaccine between June 2 and December 31 (Table I). The average age of the vaccinated staff was 41.3 years ± 7.9. There were 94 men (54%) and 80 women (46%). The vaccines administered were AstraZeneca (63%), Johnson & Johnson (36%), and Pfizer (1%). Forty-seven vaccinated staff members reported refusal by people around them to be vaccinated following adverse events, and 89 staff members (22%) reported no such behavior. The information was not applicable for 38 staff members (27%).

The main reasons for accepting vaccination were protection against the disease (n = 132; 76%), travel abroad (n = 23; 33%), and feeling obliged to do so (n = 10; 6%). Of the doctors, 62 out of 123 (50%) and of the nurses, 62 out of 251 (25%) were vaccinated. The number of vaccinated and unvaccinated staff surveyed is shown in Table I. Overall, 78% (136/174) of vaccinated workers reported adverse events after vaccination, including 89% (121/136) within 24 hours, 10% (14/136) within one week, and one person within two weeks. Of those who reported adverse events, 88 (75%) had received the AstraZeneca vaccine and 47 (35%) had received the Johnson & Johnson vaccine. No adverse events were reported by the worker who received the Pfizer vaccine.

**Table I:** Number of staff vaccinated or not surveyed according to profile from June to December 2021 at the CHU-T

Function	Total number (N)	Vaccinated, n (%)	Vaccinated respondents	Unvaccinated n (%)	Unvaccinated respondents n (% unvaccinated)
Doctors	123	62 (50)	62	61 (50)	55 (90)
Nurses	251	62 (25)	62	189 (75)	31 (16)
Pharmacists	10	5 (50)	5	5 (50)	2 (40)
Midwives	36	7 (19)	7	29 (80)	10 (34)
Ward assistants	91	9 (9)	9	82 (90)	5 (6)
Human resources officers (HR)	8	8 (100)	8	0 (0)	0 (0)
Radiology technicians	4	4 (100)	4	0 (0)	0 (0)
Physiotherapists	4	2 (50)	2	2 (50)	0 (0)
Laboratory technicians	17	3 (18)	3	14 (82)	3 (21)
IT specialists	2	2 (100)	2	0 (0)	0 (0)
Secretaries	14	3 (21)	3	11 (79)	7
Receptionists	2	1 (50)	1	0 (0)	0 (0)
Social services administrators	2	1 (50)	1	1 (50)	0 (0)
Laundry staff	6	1 (17)	1	5 (83)	0 (0)
Accountants	8	2 (25)	2	6 (75)	1 (13)
Cashiers	13	1 (8)	1	12 (92)	0 (0)
Drivers	6	1 (1)	1	5 (83)	0 (0)

Table II presents the main adverse events according to the type of vaccine received. A total of 2.3% (4 out of 174) of vaccinated individuals tested positive for COVID-19 by PCR at the CHU-T laboratory. Three of the vaccinated individuals, who received the Johnson & Johnson vaccine, developed symptoms of the disease 30, 66, and 74 days after vaccination, respectively. The agent who received the AstraZeneca vaccine tested positive 174 days after vaccination. The incidence of COVID-19 infection among unvaccinated agents is unknown.

**Table II:** Main adverse events reported according to the vaccines (Johnson & Johnson and AstraZeneca) received in individuals between June and December 2021 at the CHU-T

Adverse events	Modalities	J&J		Astra Zeneca	
n	%	n	%
Muscle soreness	Yes	22	35	66	60
	Non / No	41	65	44	40
Headaches	Yes	15	24	54	51
	Non / No	48	76	56	49
Fever	Yes	9	14	59	54
	Non / No	54	86	51	46
Pain at the injection site	Yes	23	36	46	26
	Non / No	40	63	128	74
Feeling unwell	Yes	22	35	26	24
	Non / No	41	65	84	76
Dizziness	Yes	5	8	9	8
	Non / No	58	92	101	92
Vomiting	Yes	0	0	4	4
	Non / No	0	0	106	96
Nausea	Yes	1	2	8	7
	Non / No	62	98	102	93
Epistaxis	Oui / Yes	0	0	1	1
	Non	0	0	173	99

Information on the reasons for not accepting vaccination was obtained from 134 people (29% of unvaccinated staff), 60% of whom were female. The average age of the unvaccinated staff members surveyed was 33 ± 8 years old. Regarding the profiles of the unvaccinated respondents, information was available for 126 of the 134 respondents (eight missing). Of those, there were 55 doctors (44%), 31 nurses (28%), 10 ward assistants (8%), 7 secretaries (8%), 5 midwives (4%), 4 nursing assistants (3%), 4 research nurses (3%), 3 laboratory technicians (2%), 2 pharmacists (2%), 2 state health engineering technicians (2%), 1 human resources officer (2%), 1 accounting officer (1%), and 1 other unspecified (1%).

The main reasons for not accepting vaccination, categorized by profile, are presented in Table III. Other reasons were collected but were not associated with specific profiles due to a lack of information in this field. These reasons included death after vaccination (n = 1), lack of experience in vaccine manufacturing (n = 1), vaccine not available at the peak (n = 1), reduced immunity period (n = 1), lack of information on where the vaccine was manufactured (n = 1), lack of trust in producing countries (n = 1), short pharmacovigilance period (n = 1), and lack of information on fertility in women (n = 1).

**Table III:** Reasons for not accepting COVID-19 vaccination among 126 staff members (all profiles combined) at the CHU-T from June to December 2021

Reason for not vaccinating	All profiles (N = 126)	%	Doctors and pharmacists (N = 57)	% (n /N)	Nurses + Midwives + laboratory technicians (N = 43)	% (n /N)	Other staffs (N = 26)	% (n /N)
Uncertainty about effectiveness	106	84	46	81	35	81	20	77
Uncertainty about safety	99	79	48	84	28	65	18	69
Contradictory debates among researchers about vaccines	92	73	41	72	31	72	12	46
Personal preference	89	71	40	70	24	56	21	81
Contradictory debates among researchers about the disease	79	63	34	60	28	65	15	58
Adverse events experienced by relatives	69	55	26	46	26	61	13	50
Cases reported in vaccinated individuals	54	41	11	19	28	65	13	50
Multiple vaccines	54	43	27	47	18	42	6	23
Lack of trust in the government	41	33	20	35	11	26	7	27
Low incidence in Burkina Faso	40	32	19	33	10	23	6	23
Questionable vaccine storage	38	30	17	30	11	26	7	27
Contradictory information on social media	28	22	9	16	11	26	15	58
Low mortality rate in Burkina Faso	34	27	19	33	5	12	5	19
Allergies to vaccines	23	18	6	11	13	30	4	15
Already vaccinated or ill	18	14	5	9	6	14	6	23
Disbelief in the disease	14	11	5	9	5	12	3	12
Pregnancy	9	7	1	2	3	7	4	15
Vaccines from foreign countries	37	29	15	26	11	26	9	35

Other staff: (ward assistants (n = 10), human resources staff (n = 1), accounting staff (n = 1), secretaries (n = 7), state health engineering technicians (n = 2), health research officers (n = 4), other unspecified (n = 1

## Discussion

This study allowed us to evaluate the status of the CHU-T staff's acceptance of the COVID-19 vaccine and determine the reasons for accepting or refusing it.

However, the results cannot be generalized to all healthcare facilities in Burkina Faso because the study was conducted in a single hospital that was the referral hospital for the treatment of 2020 pandemic-era patients. Another limitation is that we did not record any cases of COVID-19 among unvaccinated individuals during the study period, which could have motivated staff to get vaccinated. Furthermore, the small number of subjects interviewed about their reasons for not getting vaccinated limits the representativeness of the sample and the extrapolation of the study results. Self-administering the questionnaire could have also biased the results, as unvaccinated individuals may have misunderstood the closed-ended questions and provided inaccurate answers. Nevertheless, the results of this study provide valuable insights into the motivations for or against vaccination among hospital staff. The low proportion of vaccinated staff at the CHU-T reflects the reluctance of nearly 70% of healthcare workers. This percentage is higher than that reported by Khamis *et al.* in an analytical study on vaccine hesitancy conducted among 433 healthcare workers in Oman in the Middle East in 2021. In the context of Burkina Faso, many prejudices accompanied the COVID-19 epidemic. Some citizens believed that the disease was only affecting the wealthy because the first people diagnosed had returned from trips abroad, particularly to Europe. Others thought black Africans were immune to the disease [5, 16].

Some considered it to be a common disease, like seasonal flu, given its low incidence and mortality rate compared to what was reported on other continents [4, 12]. Healthcare capacity was often overwhelmed [3, 5], which certainly contributed to healthcare workers’ decision to get vaccinated to avoid infection.

The proportion of vaccinated healthcare workers was low, with a higher percentage of nurses than doctors getting vaccinated, even though these workers are on the front lines of the hospital response to the pandemic. They are at much higher risk of infection due to constant and sometimes prolonged contact with patients [8].

Vaccination against COVID-19 has been proposed as a control strategy, but it has been met with varying degrees of acceptance among the general population, healthcare professionals, and researchers (e.g., public health experts, immunologists, biologists, and microbiologists) around the world. In our study, the main reason for accepting vaccination was fear of infection. This reason was also identified by Paquin *et al.* in an analytical study of 342 healthcare workers in Côte d'Ivoire in 2021. Mandatory vaccination for travel was an important motivation. During the pandemic, vaccination was one of the essential requirements for entry into several countries. The common reasons for not accepting vaccination among doctors, nurses, and other healthcare workers were uncertainty about the vaccine's efficacy and safety and conflicting debates among researchers about the vaccine and the disease itself. However, adverse events experienced by a vaccinated relative and the occurrence of SARS-CoV-2 infection in vaccinated individuals were mostly reported by nurses and other healthcare workers, but not by doctors. Vaccine refusal following the dissemination of information is probably due to unreliable sources, most of which originate from social media or uncertified websites. From the outset of the epidemic, political authorities, through the Ministry of Health and the Ministry responsible for Research, should make healthcare workers aware of reliable sources of scientific information and ensure that national sources of information are institutional in origin. Proactive, rapid surveys of target populations would provide useful data for tailoring official communications to vaccine skeptics and anti-vaccine advocates, and would help to better understand their concerns and information needs. Additionally, awareness-raising, training, and prevention strategies should be tailored to different groups.

Other authors have described the reasons for not vaccinating, such as concerns about efficacy and safety [2, 16]. Touré *et al.* described fear of adverse events, uncertainty about the lack of transparency in clinical trials and research protocols, and doubts about vaccine efficacy in a descriptive study of 303 users of two healthcare facilities in the municipality of Youpougon in Côte d'Ivoire [18]. Touré *et al.* also described fear of an adverse effect of vaccines on pregnancy or the ability to become pregnant.

Minor adverse events, such as headaches, pain at the injection site, and fever, are frequently experienced and described in several studies and indicate a normal immune response [14]. We did not observe any serious adverse events among vaccine recipients. This could be explained by the cross-sectional nature of our survey and the small size of our study population.

Three out of 110 people (2.7%) who received the Johnson & Johnson vaccine and one out of 63 (1.6%) of those who received the AstraZeneca vaccine tested positive for minor signs of COVID-19. Other authors have documented cases of COVID-19 following vaccination [1, 17].

## Conclusion

This study allowed us to evaluate the acceptance of the COVID-19 vaccine among our hospital staff, determine the reasons for acceptance and non-acceptance, and review adverse events reported following vaccination. However, the proportion of healthcare workers who agreed to be vaccinated is low among frontline workers involved in the management of the current pandemic but also other infectious diseases. These workers need interventions to increase their participation.

## Funding

This study received no funding.

## Acknowledgments

We would like to thank Dr. Souleymane Kaboré, who made significant contributions to this study but passed away after the final version of the manuscript was completed. We pay tribute to him.

## Contributions of Authors

Noélie Zoungrana-Yameogo designed the study. She participated in data collection and performed data analysis and interpretation. Dr. Souleymane Kabore participated in writing the final manuscript. Drs. David Kangoye, Issa Ouedraogo, Yassia Bamogo, Abdoulaye So, Arielle Rita Belem, and Alban Michel Bassolé read and provided comments on the final version of the manuscript. Professor Idrissa Sanou approved publication of the manuscript.

## Conflict of interest

The authors declare no conflict of interests

## Appendix 1: Questionnaire for vaccinated staff

Hello,

I am Dr.__. We would like to ask you some questions about the status of your vaccination against the SARS-CoV-2 virus as part of a study aimed at assessing the vaccination status of CHU-T staff. We obtained your contact information from the registry of vaccinated staff members. The results will be published anonymously, and your participation is optional.

**1. General Information:**

Age: /__/ years old Gender: M /__/ F /__/

Qualification: Doctor / / Pharmacist / / Nurse / / Laboratory technician / / Radiology Technician / /

Nursing Assistant / / Ward Assistant / / Midwife / /Human Resources / /Agent Communicator / /Driver / /

Laundry Worker /__/Security Guard /__/ IT Specialist /__/ Physiotherapist /__/ Secretary Other /__/ If other, please specify……

**2. What motivated you to get vaccinated?**

Fear of getting sick / / feeling obligated / / example of a colleague / / Requirement from a superior /__/

Example set by a superior /__/ Contribution to science /__/ Example set by a family member /__/ Travel /__/

Other /__/ If other, please specify /__/

**3. Type of vaccine:**

Type of vaccine received: AstraZeneca /__/ Johnson & Johnson /__/ Other /__/

**4. Did you experience any adverse events after vaccination?**

Yes /__ No /__

If yes:

1) Headache: Yes /__No /__

2) Fever: Yes /__/ No /__/

3) Muscle aches: Yes /__/ No /__/

4) Pain at the injection site: Yes /__/ No /__/

5) Redness: Yes /__No /__

6) Dizziness: Yes /__/ No /__/

7) Feeling unwell: Yes / No

8) Nausea: Yes /__/ No /__/

9) Vomiting: Yes /__/ No /__/

10) Thrombosis: Yes /__/ No / /

11) Myocarditis: Yes /__/ No / / Other: /__/

**5. How long did it take for the adverse events to appear?**

Immediately /__/ Days /__/ Months /__/

**6. How did those around you react to the adverse events?**

Refusal to be vaccinated /__No reaction /__NA if no side effects /__

## Appendix 2: Questionnaire for unvaccinated agents (volunteers)

Hello,

I am Dr. …, we would like to gather information on the reasons for not getting vaccinated against COVID-19 from unvaccinated staff at the CHU-T. Your participation in this survey by completing this form is entirely voluntary, your data will remain anonymous, and the results will be presented anonymously.

Thank you for your participation.

**1. General information**

Age: /__/ years old Gender: M /__/ F /__/

Qualification: Doctor /__/ Pharmacist /__/ Nurse /__/ Laboratory technician /__/

State-registered radiology technician /__/ Nursing assistant /__/ Ward assistant /__/ Midwife /__/

Communicator /__/ Driver /__/ Laundry worker /__/ Security guard /__/ IT technician /__/

Electrician /__/ HR agent /__/ Physiotherapist /__/ Secretary /__/ DFC agent /__/

Other /__/ If other, please specify__

**2. Reasons for not being vaccinated, multiple choices possible**

1) Uncertainty about effectiveness Yes /__/ No /__/

2) Uncertainty about safety Yes /__/ No /__/

3) Multiple vaccines Yes /__/ No /__/

4) Disbelief in the disease Yes /__/ No /__/

5) Low incidence (not many cases in Burkina Faso) Yes /__/ No /__/

6) Low mortality rate (not many deaths in Burkina Faso) Yes /__/ No /__/

7) Adverse events experienced by a relative Yes /__/ No /__/

8) Vaccines from other sources Yes /__/ No /__/

9) Contradictory debates among researchers about the disease Yes /__/ No /__/

10) Contradictory debates among researchers about vaccines Yes /__/ No /__/

11) Doubts about vaccine storage (storage temperature) Yes /__/ No /__/

12) Vaccine allergies Yes /__/ No /__/

13) Pregnancy Yes /__/ No /__/

14) Already had COVID-19 Yes /__/ No /__/

15) Conflicting information on social media Yes /__/ No /__/

16) Distrust of the government Yes /__/ No /__/

17) Personal preference Yes /__/ No /__/

18) Reported cases of COVID-19 in people who have already been vaccinated Yes /__/ No /__/

19) Other, please specify…….
